# Uncovering High-dimensional Structures of Projections from Dimensionality Reduction Methods

**DOI:** 10.1016/j.mex.2020.101093

**Published:** 2020-10-10

**Authors:** Michael C. Thrun, Alfred Ultsch

**Affiliations:** a Dept. of Hematology, Oncology and Immunology, Philipps-University of Marburg, Baldingerstraße, D-35043 Marburg; b Databionics Research Group, Philipps-University of Marburg, Hans-Meerwein-Straße 6, Marburg D-35032, Germany

**Keywords:** Dimensionality reduction, Projection methods, Data visualization, Unsupervised neural networks, Self-organizing maps

## Abstract

Projections are conventional methods of dimensionality reduction for information visualization used to transform high-dimensional data into low dimensional space. If the projection method restricts the output space to two dimensions, the result is a scatter plot. The goal of this scatter plot is to visualize the relative relationships between high-dimensional data points that build up distance and density-based structures. However, the Johnson–Lindenstrauss lemma states that the two-dimensional similarities in the scatter plot cannot coercively represent high-dimensional structures. Here, a simplified emergent self-organizing map uses the projected points of such a scatter plot in combination with the dataset in order to compute the generalized U-matrix. The generalized U-matrix defines the visualization of a topographic map depicting the misrepresentations of projected points with regards to a given dimensionality reduction method and the dataset.

• The topographic map provides accurate information about the high-dimensional distance and density based structures of high-dimensional data if an appropriate dimensionality reduction method is selected.
• The topographic map can uncover the absence of distance-based structures.
• The topographic map reveals the number of clusters in a dataset as the number of valleys.

## Graphical abstract

**Figure:**
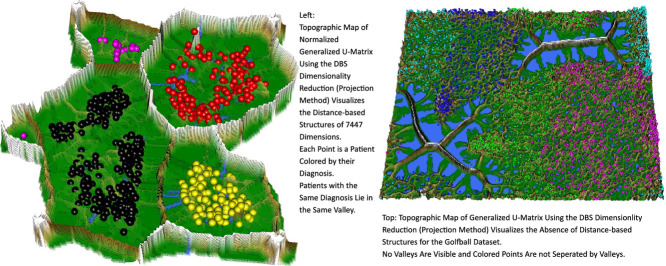

Specifications table

**Table:** 

Subject Area	*• Computer Science*
More specific subject area:	*Unsupervised Machine Learning*
Method name:	*Topographic Map Generation Using the Generalized Umatrix*
Name and reference of original method	*Thrun, M. C., & Ultsch, A.: Swarm Intelligence for Self-Organized Clustering, Journal of Artificial Intelligence*, in press, doi:10.1016/j.artint.2020.103237,*2020.* *Ultsch, A., Thrun, M.: Credible Visualizations for Planar Projections, Proc. Workshop on Self-Organizing Maps (WSOM), pp. 256–260, Nancy, 2017.* *Thrun, M. C., Lerch, F., Lötsch, J., Ultsch, A.: Visualization and 3D Printing of Multivariate Data of Biomarkers, In: Skala, V. (Ed.): 24th Conf. on Computer Graphics, Visualization and Computer Vision, Plzen, pp.* 7*–*16*2016.* *Ultsch, A.: Maps for the Visualization of high-dimensional Data Spaces, Proc. Workshop on Self organizing Maps (WSOM), pp. 225–230, Kyushu, Japan, 2003.* *Ultsch, A., Siemon, H.P.: Kohonen's Self Organizing Feature Maps for Exploratory Data Analysis, In Proceedings Intern. Neural Networks, Kluwer Academic Press, Paris, pp. 305–308, 1990.*
Resource availability	https://CRAN.R-project.org/package=GeneralizedUmatrix

This article is divided into four parts. The first describes the unsupervised artificial network of self-organizing maps (SOMs) in general; the second part describes the application of simplified emergent self-organizing maps (sESOM) for projection methods and the third part describes the visualization of the topographic map with hypsometric tints based on the output of sESOM. The last part shows the application in three examples. The method is part of [30]; it is a co-submission of that article (ARTINT_103237) and the method's description originates from several sections of the Ph.D. thesis, “Projection-Based Clustering through Self-Organization and Swarm Intelligence” [27].

## Emergent self-organizing map (ESOM)

Self-organizing (feature) map (SOM) was invented by [13],[14] and is a type of unsupervised neural learning algorithm. In contrast to other neural network models^1^ a SOM consists of an ordered two-dimensional layer of neurons called units. Neurons are interconnected nerve cells in the human neocortex [H. [25], p. 22], and the SOM approach was inspired by somatosensory maps (e.g. see [[9], p. 421] cites [8], see also [[12], p. 335]). There are two types of SOM algorithms: online and batch [7]. The first is stochastic, whereas the second is deterministic, which means that it yields reproducible results for a given parameter setting. However, Fort et al. have argued “that randomness could lead to better performances” [[7], p. 12].

The main differences between batch-SOM [16] and online-SOM [15] lie in the updating and averaging of the input data. In batch-SOM, prototypes (see Eq. (1) below) are assigned to the data points and the influences of all associated data points are calculated simultaneously, in contrast to online-SOM, in which sequential training of the neurons is applied (as described in detail below). The batch-SOM method has been shown to produce topographic mappings of varying quality depending on the pre-defined parametrization [7], and “the representation of clusters in the data space on maps trained with batch learning is poor compared to sequential training“ [21]. An important comparison between the batch-SOM approach and ant-based clustering was presented by [10] and will be elaborated upon in chapter 7. No objective function is used in online-SOM [[17], p. 241], and SOM remains a reference tool for two-dimensional visualization [[17], p. 244].

In one common approach to applying the SOM concept, the algorithm acts as an extension of the k-means algorithm [4] or is a partitioning method of the k-means type [20]. In such a case, only a few units are used in the SOM algorithm to represent the data [23], which results in direct clustering of the data. Here, each neuron can be considered to represent a cluster. For example, Cottrell and de Bodt used 4 × 4 units to represent the 150 data points in the Iris dataset ([Ultsch et al., 2016a] cites [3]). Therefore, the conventional SOM algorithm is called k-means-SOM here. This SOM algorithm also has two common extensions called Heskes-SOM [11] and Cheng-SOM; these two extensions include objective functions [1] and are not discussed further in this thesis. The optimization of objective functions in general will be discussed in chapter 6, where it will be argued that it is not useful for the goal of this thesis. Chapter 7 will show that objective functions are incompatible with self-organization.

The other approach to applying SOM is to exploit its emergent phenomena through self-organization, in which case it is necessary to use a large number of neurons (>4000) [31]. This enhancement of the online-SOM approach is called emergent SOM (ESOM). In such a case, the neurons serve as a projection of the high-dimensional input space instead of a clustering, as is the case in k-means-SOM.

Let $M\;=\;\{m_{1},\;\ldots ,\;m_{n}\}$be the positions of *n* neurons on a two dimensional lattice^2^ (feature map) and $W\;=\;\{w\left(m_{i}\right)\;=w_{i}\;|i\;=\;1,\;\ldots \;n\}$the corresponding set of weights or prototypes of *n* neurons, then, the SOM training algorithm constructs a non-linear and topology-preserving mapping of the input space *I* by finding the best matching unit (bmu) for each *l* ∈ *I*:

(1) $$bmu\left(l\right)=\underset{m_{i}\in M}{\text{argmin}}\left\{D\left(l,w_{i}\right)\right\},\;i\in \left\{1,\ldots ,n\right\}$$

if in Eq. (1) a distance in the input space *I* between the point *l* and the prototype *w_i_* is denoted.

In each step, SOM learning is achieved by modifying the prototypes (weights) in a neighborhood in Eq. (2).

(2) $$\Delta w\left(R\right)=\eta \left(R\right)*h\left(bmu\left(l\right),m_{i},R\right)*\left(l-w\left(m_{i}\right)\right)$$

The cooling scheme is defined by the neighborhood function $h:M\times M\times R^{+}\to \left[-1,1\right]$and the learning rate *η*: $R^{+}\to \left[0,1\right]$, where the radius *R*decreases until R=1in accordance with the definition of the maximum number of epochs. In contrast to all previously introduced projection methods, no objective function is used in the ESOM algorithm. Instead, ESOM uses the concept of self-organization (see chapter 6 for further details) to find the underlying structures in data.

The structure of a (feature) map is **toroidal**; i.e., the borders of the map are cyclically connected [31], which allows the problem of neurons on borders and, consequently, boundary effects to be avoided. The positions *m* ∈ *M* of the BMUs exhibit no structure in the input space [31]. The structure of the input data emerges only when a SOM visualization technique called U-matrix is exploited [34].

Let *N*(*j*) be the eight immediate neighbors of *m_j_*  ∈  *M*, let *w_j_* ∈ *W* be the corresponding prototype to *m_j_*, then the average of all distances between prototypes *w_i_*

(3) $$u\left(j\right)=\frac{1}{n}\sum_{i\in N\left(j\right)}D\left(w\left(m_{i}\right),w\left(m_{j}\right)\right),\;n=\left|N\left(j\right)\right|$$

A display of all U-heights in Eq. (3) is called a U-matrix [34].

The U-matrix technique that is generally applicable for all projection methods and can be used to visualize both distance- and density-based structures [27],[35]. This visualization technique is the further development of the idea that the U-matrix can be applied to every projection method [33].

In this work, the visualization technique results in a topographic 3D landscape. Here, the requirements are a heavily modified emergent self-organizing map (ESOM) algorithm and a method of high-dimensional density estimation. Contrary to [33], the process of computing the resulting topographic map is completely free of parameter dependence and accessible by simply by downloading the corresponding R package [Thrun/Ultsch, 2017b].

## Simplified ESOM

To calculate a U*-matrix for any projection method, a modified ESOM algorithm is required. The first step is the computation of the correct lattice size.

On the x axis, let the lattice begin at 1 and end at a maximal number denoted by Columns C (equal to the number of columns in the lattice); similarly, on the y axis, let the lattice begin at a maximal number denoted by Lines L and end at 1. Then, the first condition is expressed as

(I) $$\frac{L-1}{C-1}\approx \frac{\left|\max\left(y\right)-\min\left(y\right)\right|}{\left|\max\left(x\right)-\min\left(x\right)\right|}=\frac{dy}{dy}=\Delta$$

The second condition is that the lattice size should be larger than NN^3^:

(II) L*C≥NN

The first condition (I.) implies that the lattice size should be as close to equal to the size of the coordinate system as possible. The second condition (II.) is required for emergence in our algorithm (for details, see [31]). The resulting equation to be solved is Eq. (4)

(4) $$L^{2}+L\left(1+\Delta \right)-\text{NN}*\Delta \geq 0$$

which yields Eq. 5:

(5) $$L\geq -\frac{1+\Delta }{2}+\sqrt{{\left(\frac{1+\Delta }{2}\right)}^{2}+\text{NN}*\Delta }$$

After the transformation from the projected points^4^ *p* ∈ *O* to points on a discrete lattice, the points are called the best-matching units (BMUs) $bmu\in B\;\subset R^{2}$of the high-dimensional data points j, analogous to the case for general SOM algorithms with *fgrid*: *O* → *B*, *p*↦ *bmu,* where *fgrid* is surjective when conditions (i) and (ii) are met.

To develop the algorithm illustrated in Listing 1, the idea of [33], in which it was suggested to “apply Self-Organizing Map training without changing the best match[ing unit] assignment”, was adopted. However, in contrast to [33], here, the transformation *fgrid* is defined precisely to calculate the BMU positions and the structure of the lattice is toroidal; i.e., the borders of the lattice are cyclically connected [31].

**Listing 1:**
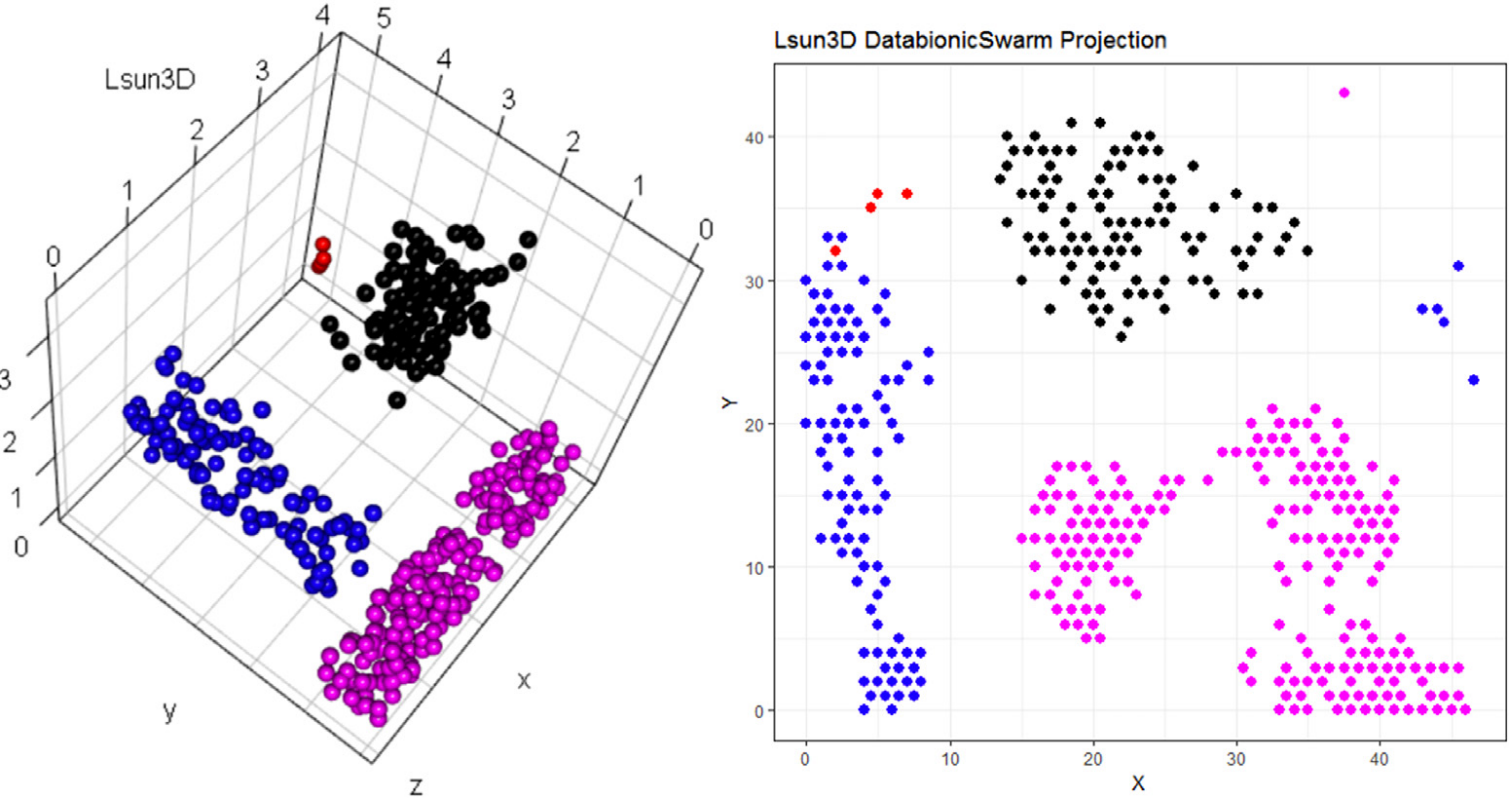
Lsun3D data set and DBS projection [Thrun/Ultsch, 2020]. The projection does not indicate which projected points are similar to each other with regards to the high-dimensional structures. The projected points at the top are in Figure 2 at the bottom on the projected points on the left are in Figure 2 on the right.

Based on the relevant symmetry considerations,^5^ a simplified version of ESOM (sESOM) is introduced here. No epochs or learning rate are required, because the cooling scheme is defined by a special neighborhood function $h:M\;\times \;M\;\times R^{+}\to \left[0,1\right]$.

Let $M=\{m_{1},\ldots ,m_{n}\}$be a set of neurons (where *m_i_* are the lattice positions) with the corresponding prototype set $W=\{w_{1},\ldots ,w_{n}\}$, where dim(W)=dim(I) and #*W*=#M; then, the neighborhood function h is defined in Eq. (6):

(6) $$h=\{\begin{array}{c} 1-\frac{d{\left(j,l\right)}^{2}}{\pi R^{2}},\;iff\;\frac{d{\left(j,l\right)}^{2}}{\pi R^{2}}<1\\ 0,\;else \end{array}$$

In sESOM, learning is achieved in each step by modifying the weights in a neighborhood in Eq. (7):

(7) $$\Delta w\left(R\right)=1*h\left(bmu\left(j\right),m_{i},R\right)*\left(j-w\left(m_{i}\right)\right)\;$$

In contrast to [33], the algorithm does not require any input parameters, and the resulting visualization is not a two-dimensional gray-scale map but rather a topographic map with hypsometric tints [28]. The entire algorithm is summarized in Listing 1.

## Topographic map with hypsometric

The U*-matrix visualization technique produces a topographic map with hypsometric tints [28]. Hypsometric tints are surface colors that represent ranges of elevation [22]. Here, a specific color scale is combined with contour lines.

The color scale is chosen to display various valleys, ridges and basins: blue colors indicate small distances (sea level), green and brown colors indicate middle distances (low hills), and shades of white colors indicate large distances (high mountains covered with snow and ice). Valleys and basins represent clusters, and the watersheds of hills and mountains represent the borders between clusters (Fig. 2 and Fig. 6).

The landscape consists of receptive fields, which correspond to certain U*-height intervals with edges delineated by contours. This work proposes the following approach (see [[28], p. 10]): First, the range of U*-heights is split up into intervals, which are assigned uniformly and continuously to the color scale described above through robust normalization [18]. In the next step, the color scale is interpolated based on the corresponding CIELab color space [2]. The largest possible contiguous areas corresponding to receptive fields in the same U*-height intervals are outlined in black to form contours. Consequently, a receptive field corresponds to one color displayed in one particular location in the U*-matrix visualization within a height-dependent contour. Let u(j) denote the U*-heights, and let q01 and q99 denote the first and 99-th percentiles, respectively, of the U*-heights; then, the robust normalization of the U*-heights u(j) is defined by Eq. (8):

(8) $$u\left(j\right)=\frac{u\left(j\right)-q01}{q99-q01}$$

The number of intervals in is defined by Eq. (9):

(9) $$\frac{1}{in}=\frac{q01}{q99}$$

The resulting visualization consists of a hierarchy of areas of different height levels represented by corresponding colors (see Figure). To the human eye, the visualization using the generalized U-matrix tool is analogous to a topographic map; therefore, one can visually interpret the presented data structures in an intuitive manner. In contrast to other SOM visualizations, e.g., [26], this topographic map presentation enables the layman to interpret sESOM results.

The use of a toroidal map for sESOM computations necessitates a tiled landscape display in the interactive generalized U-matrix tool [29], which means that every receptive field is shown four times. Consequently, in the first step, the visualization consists of four adjoining images of the same generalized U-matrix [32] (the same is true for the U*-matrix). To obtain the 3D landscape (island^6^), a shiny application can be used to cut an island out.

## Summary

**Fig. 1:**
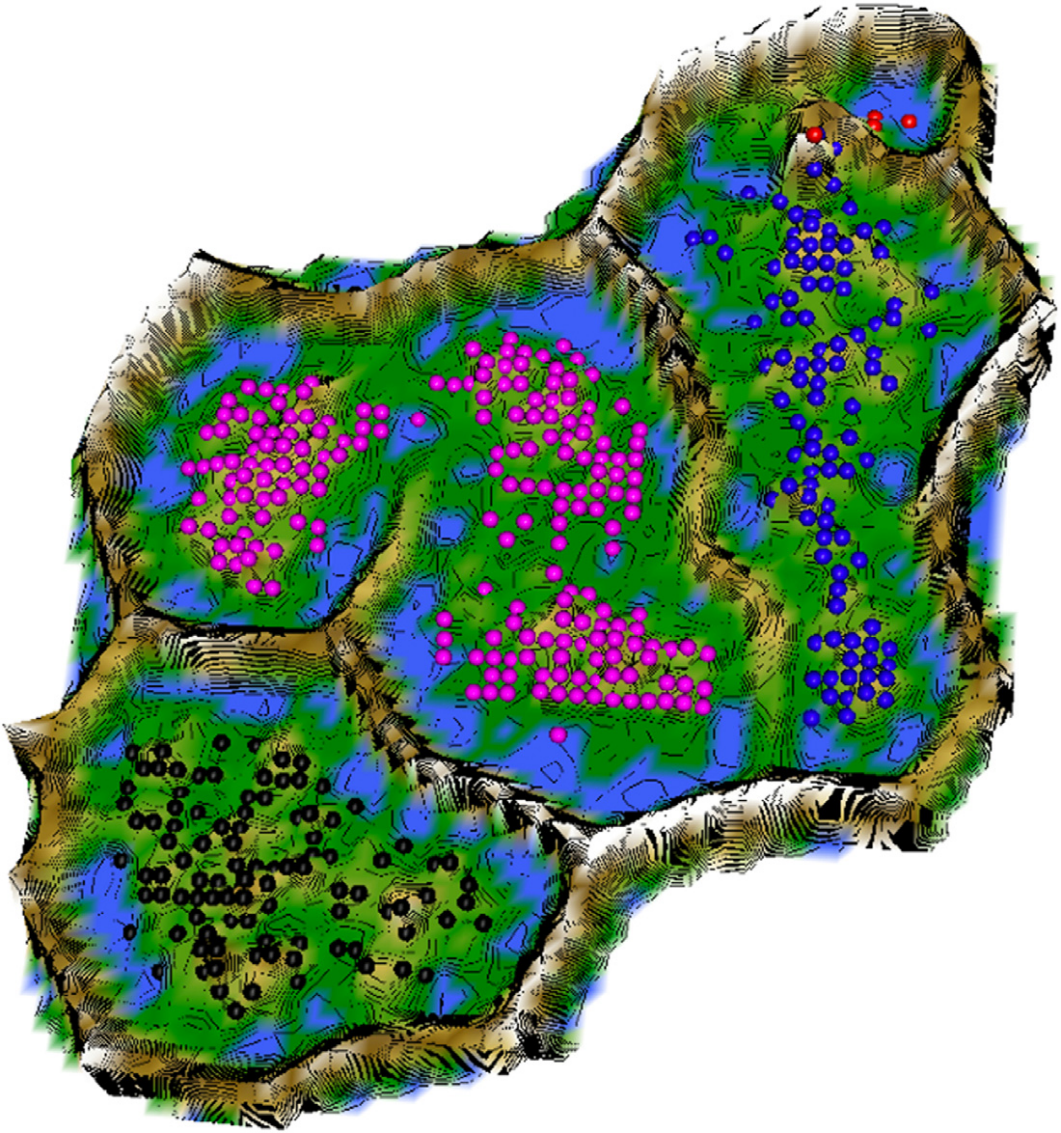
Topographic map of the projection shows a high-dimensional structures which corresponds to the prior classification of Lsund3D. The four Outliers, labeled in red, lie either on a volcano or a high mountain. The borders of the scatter plot in Figure 1 (right), which divide the blue cluster in two parts, are ignored.

**Fig. 2:**
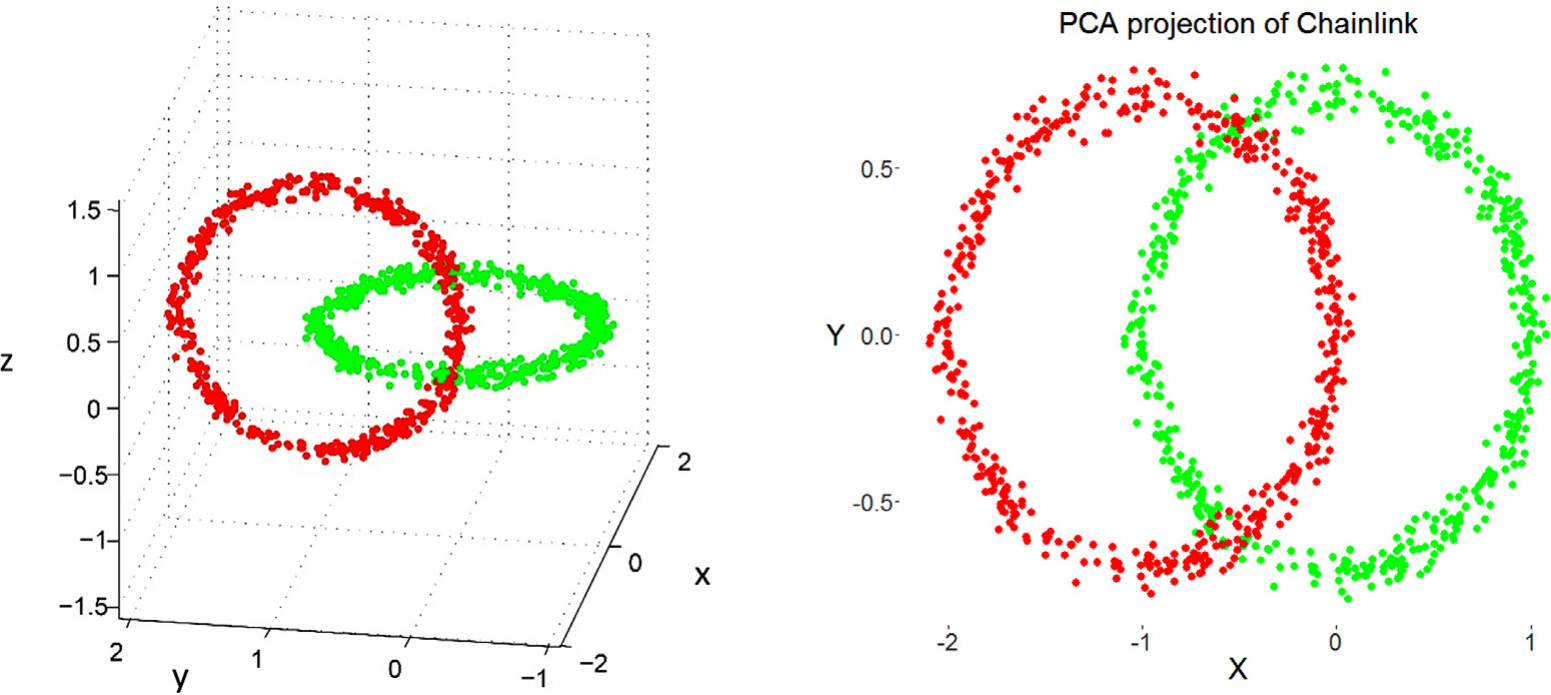
Chainlink data set (right) and PCA projection. The projection suffers from local errors in two small areas around a low number of points, but the projection is unable to visualize them.

**Fig. 3:**
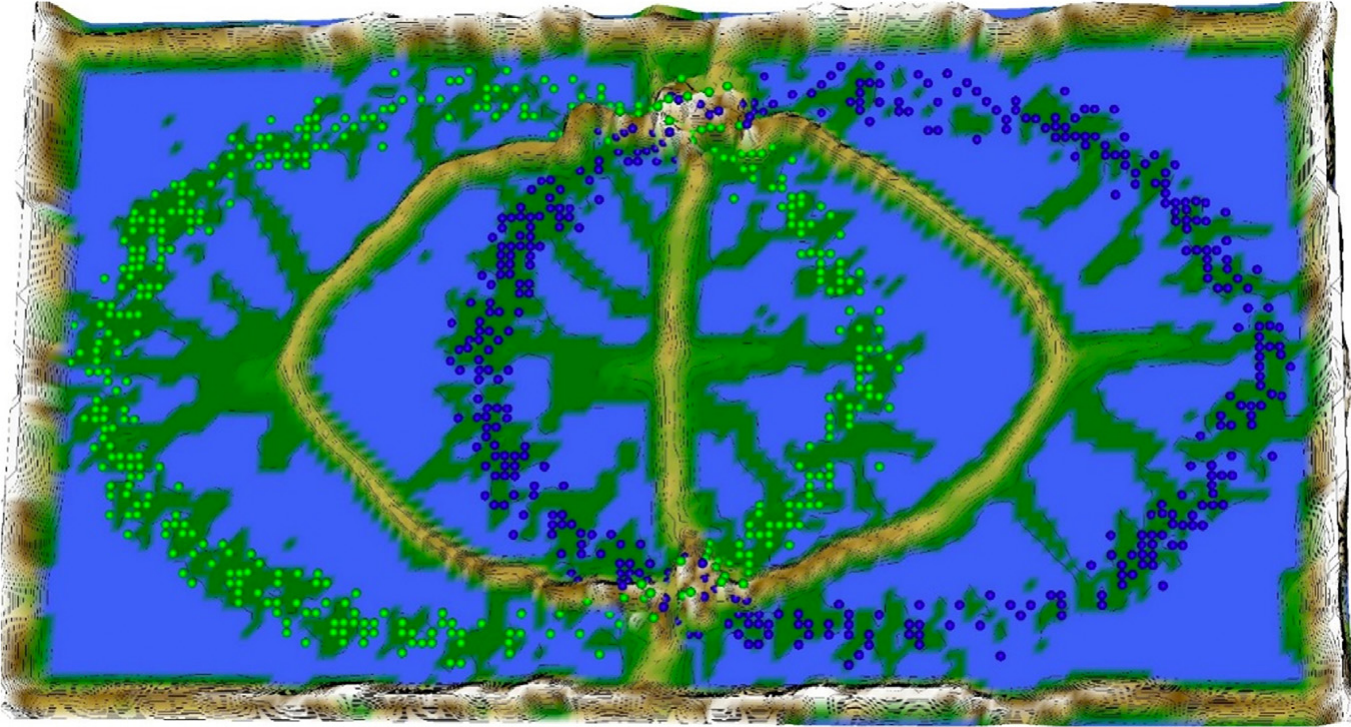
Topographic map of the PCA projection of the Chainlink data set. The errors of the projection are clearly visible. Contrary to Figure 1 and 2, the borders of the scatter plot are visualized as hills in the topographic map showing that points on the borders are far away from each other.

**Fig. 4:**
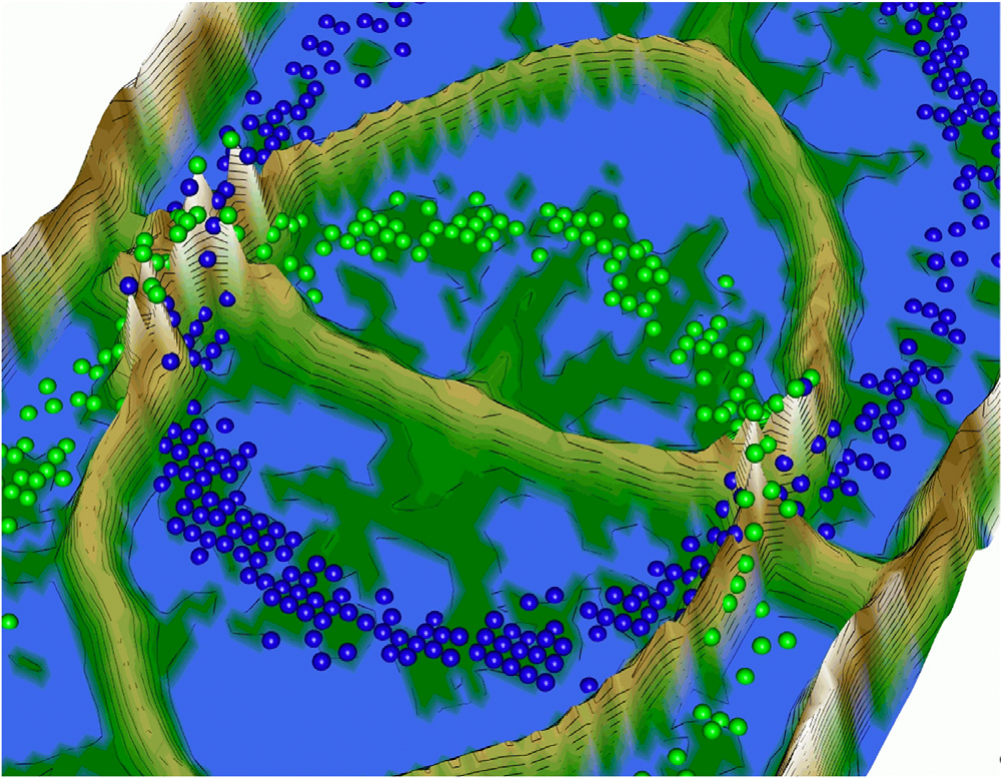
Zoomed-in view of the misrepresentation of the relative relationship between the high-dimensional points (structures) of the Chainlink dataset in the PCA projection.

**Fig. 5:**
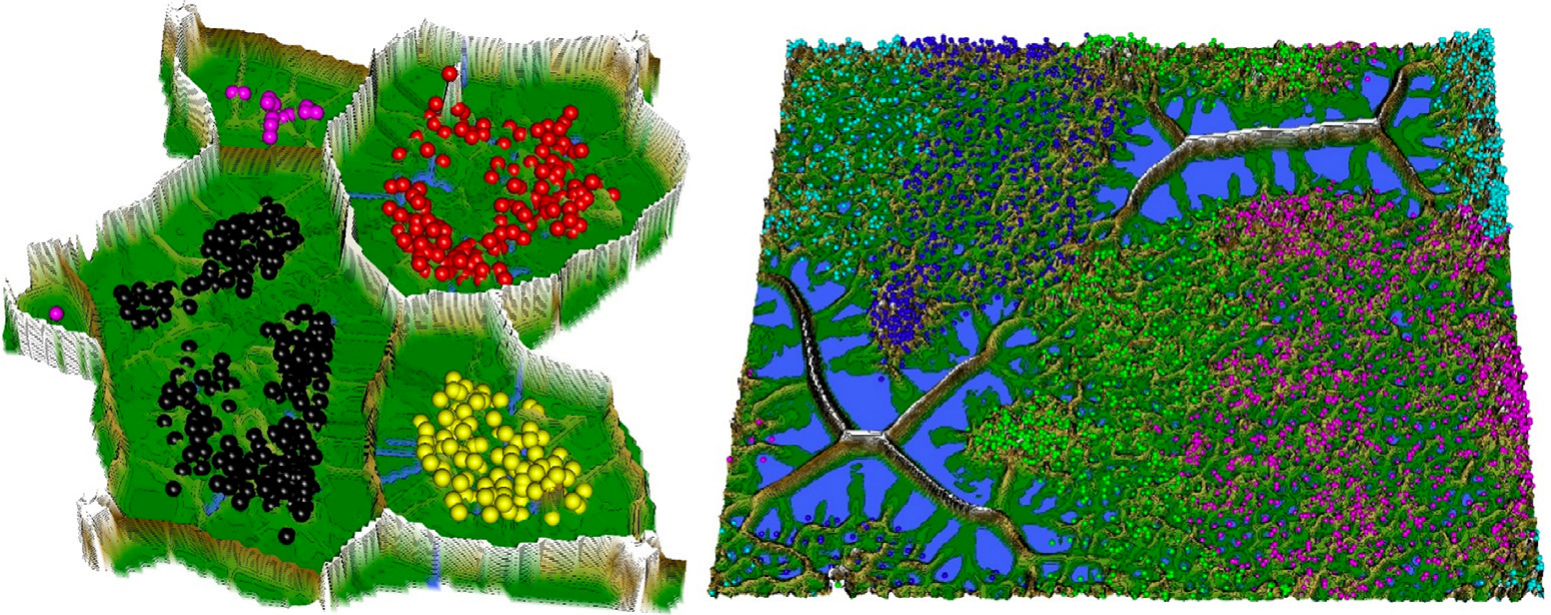
Left: topographic map of the normalized generalized U-matrix using the DBS projection module [Thrun/Ultsch, 2020] visualizes the distance-based structures of 7447 dimensions. Each point represents a patient colored by their diagnosis. Patients with the same diagnosis lie in the same valley. Right: topographic map of generalized U-matrix using the DBS projection module [Thrun/Ultsch, 2020] visualizes the absence of distance-based structures for the Golfball dataset (see Data in Brief article). No valleys are visible and colored points are not separated by watersheds of hills and mountain ranges.

**Fig. 6:**
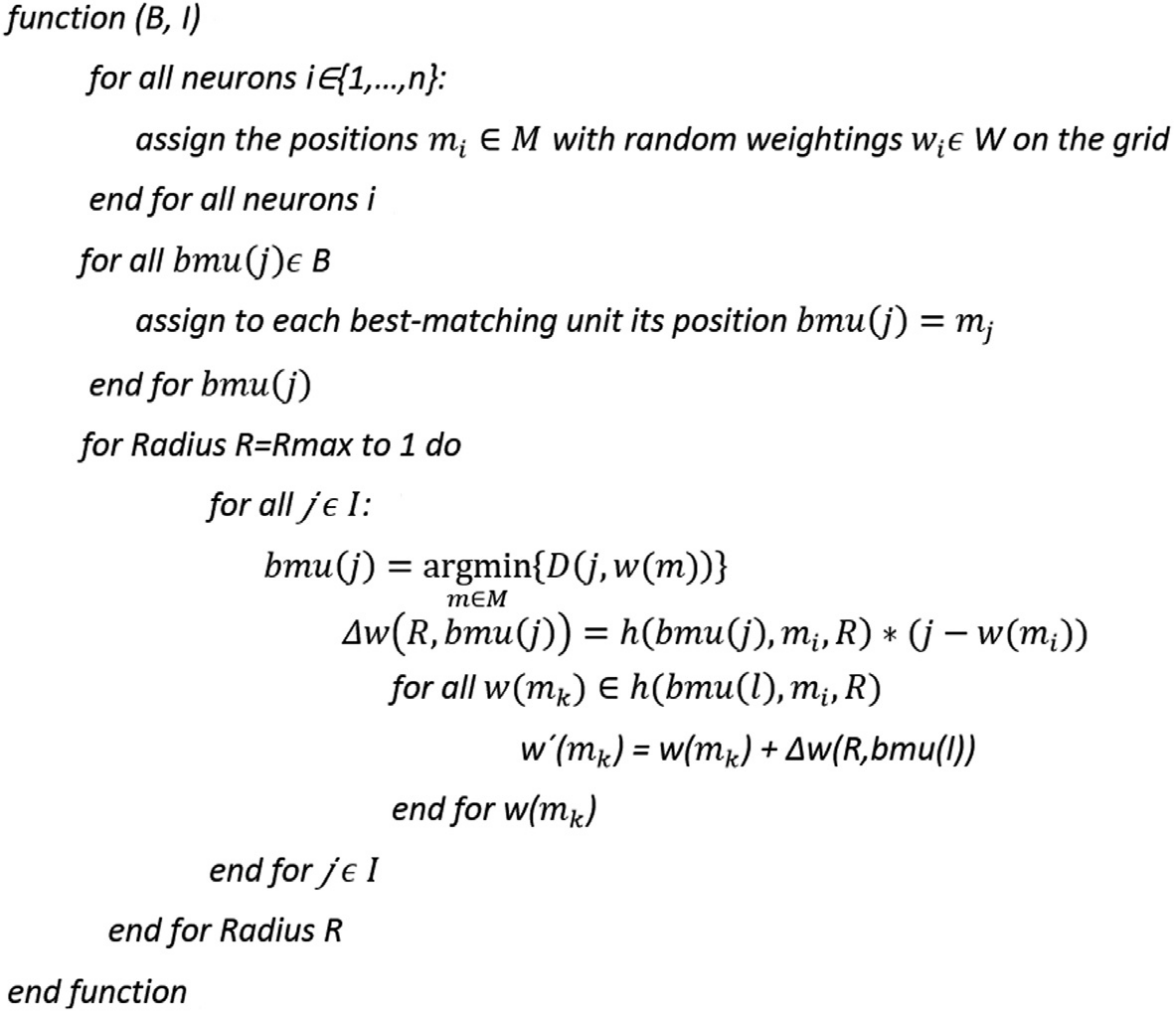
Listing 1: sESOM pseudocode algorithm implements a stepwise iteration from the maximum radius Rmax which is given by the lattice size (Rmax = C/6) stepwise with one per step and down to 1. w´(m_k) indicates that the prototype w(m_k) of neuron m_k is modified by Eq. 7 Additionally, the search for a new best matching unit still is used and these prototypes may change during one iteration. The predefined prototypes are reset to the weights of their corresponding high-dimensional data points after each iteration.

Restricting the Output space of a Projection method results in projection errors because the two-dimensional similarities in the scatter plot cannot coercively represent high-dimensional distances. This is stated by the Johnson–Lindenstrauss lemma [5] and visualized in two examples. Fig. 1 and Fig. 3 show, how scatter plots lead to misleading interpretation of the high-dimensional structures. However, scatter plots of projection methods remain the state of the art in cluster analysis as a visualization of distance and density-based structures (e.g., [[6], pp. 31–32; [19], p. 25; G. [24], p. 223; [9], pp. 119–120, 683–684]). Thus, the projected points of such a scatter plot are used in a simplified emergent self-organizing map in order to compute a generalized U-matrix. This generalized U-matrix defines the visualization of a topographic map which provides more accurate information about the high-dimensional distance and density based structures of the data. The topographic maps of the three examples are visualized in Fig. 1, Fig. 1, Fig. 2, Fig. 3, Fig. 4, Fig. 5. For Fig. 6 the projections are not shown.

Example 1: Linear Separable Structures
Example 2: Linear Non-Separable Structures
Example 3: High-Dimensional Structures versus the Absence of Such Structures

## Declaration of Competing Interest

None.

## Footnotes

1 For an overview, see [H. [25]], for deep learning see [Goodfellow et al., 2016].

2 In general this work uses the term grid if the resulting tiling is hexagonal and lattice if the resulting tiling is rectangular (see connected graph). In the context here the distinction is not important, therefore we use the term (feature) map.

3 In [31] the minimum number of 4096 neuros was proposed.

4 Or DataBot positions on the hexagonal grid of Pswarm (see [30]).

5 See [30] for details.

6 An island can be also cut interactively (or the cutting may be improved) and thus may not be rectangular
